# Unlocking the secrets: the power of methylation-based cfDNA detection of tissue damage in organ systems

**DOI:** 10.1186/s13148-023-01585-8

**Published:** 2023-10-19

**Authors:** Lijing Zhang, Jinming Li

**Affiliations:** 1 National Center for Clinical Laboratories, Institute of Geriatric Medicine, Chinese Academy of Medical Sciences, Beijing Hospital/National Center of Gerontology, No. 1 Dahua Road, Dongdan, Beijing, 100730 People’s Republic of China; 2 Peking Union Medical College, Chinese Academy of Medical Sciences, Beijing Hospital, Beijing, People’s Republic of China; 3 Beijing Engineering Research Center of Laboratory Medicine, Beijing, People’s Republic of China

**Keywords:** Methylation, cfDNA, Tissue and organ damage, Biomarkers

## Abstract

**Background:**

Detecting organ and tissue damage is essential for early diagnosis, treatment decisions, and monitoring disease progression. Methylation-based assays offer a promising approach, as DNA methylation patterns can change in response to tissue damage. These assays have potential applications in early detection, monitoring disease progression, evaluating treatment efficacy, and assessing organ viability for transplantation. cfDNA released into the bloodstream upon tissue or organ injury can serve as a biomarker for damage. The epigenetic state of cfDNA, including DNA methylation patterns, can provide insights into the extent of tissue and organ damage.

**Content:**

Firstly, this review highlights DNA methylation as an extensively studied epigenetic modification that plays a pivotal role in processes such as cell growth, differentiation, and disease development. It then presents a variety of highly precise 5-mC methylation detection techniques that serve as powerful tools for gaining profound insights into epigenetic alterations linked with tissue damage. Subsequently, the review delves into the mechanisms underlying DNA methylation changes in organ and tissue damage, encompassing inflammation, oxidative stress, and DNA damage repair mechanisms. Next, it addresses the current research status of cfDNA methylation in the detection of specific organ tissues and organ damage. Finally, it provides an overview of the multiple steps involved in identifying specific methylation markers associated with tissue and organ damage for clinical trials.

**Summary:**

This review will explore the mechanisms and current state of research on cfDNA methylation-based assay detecting organ and tissue damage, the underlying mechanisms, and potential applications in clinical practice.

## Introduction

Detecting organ and tissue damage is crucial for timely diagnosis and effective treatment of various diseases and conditions. Early detection of tissue damage can allow for prompt intervention, which can prevent further damage and potentially improve outcomes [1, 2]. Monitoring disease progression can also help inform treatment decisions and allow for adjustments as necessary [3, 4]. Additionally, evaluating treatment efficacy can help determine if interventions are effective and inform decisions about continuing or modifying treatment plans [5, 6]. Finally, in transplantation medicine, detecting tissue damage is critical for assessing the viability of organs for transplantation and ensuring successful outcomes for recipients [7, 8]. In previous research, histopathological examination of tissue slices has often been considered the “gold standard” for detecting tissue and organ damage [9]. However, this method is highly invasive and is not suitable for the early screening of tissue and organ damage.

CpG sites are a specific region on DNA, named after the presence of a C (cytosine) and a guanine nucleotide, connected by a phosphodiester bond. These sites are widely distributed throughout the human genome and are crucial for gene expression and genome stability [10–12]. In numerous studies, the methylation status of CpG sites has been closely associated with the onset and development of human diseases. DNA methylation is also altered in response to tissue damage [13]. Methylation-based assays offer a promising approach for detecting organ and tissue damage [14]. Epigenetic analysis, based on methylation patterns in DNA, involves the study of DNA methylation patterns to detect signs that may reflect tissue damage or disease-related changes. These testing methods possess a high degree of specificity and sensitivity, and can be applied to various sample types, including blood, urine, and tissue biopsies, facilitating non-invasive data collection and greatly advancing early diagnosis of tissue damage [15–17]. Methylation-based assays are being investigated for a range of applications, from early detection and monitoring of disease progression to evaluating treatment efficacy and assessing organ viability for transplantation [18, 19].

**Fig. 1:**
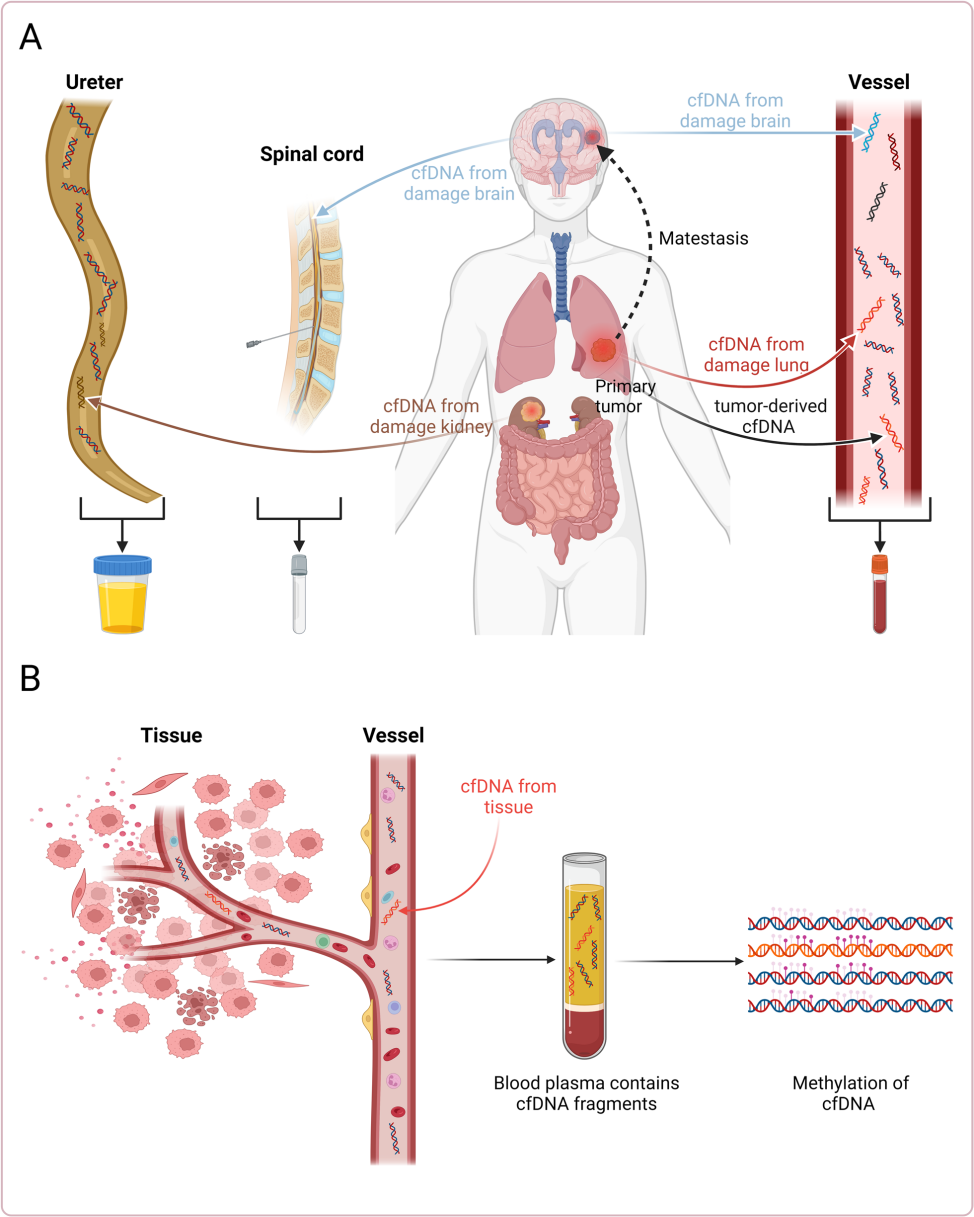
Sources of cfDNA. **A** The release of cfDNA into body fluids originates from tissues and organs, including blood, cerebrospinal fluid, and urine, among others. **B** Injured tissues and organs release cfDNA with altered methylation patterns into the bloodstream, which can be detected

Liquid biopsy, especially circulating cfDNA (cell-free DNA) methylation analysis in plasma, may become a promising non-invasive diagnostic method in tissue damage detection [20]. The collection of blood or body fluid samples is a non-invasive procedure that does not require tissue resection or tissue biopsy, thus avoiding the distress and risks of traditional tissue examination [21]. The majority of cfDNA fragments range from 80 to 200 bp with a length corresponding to nucleosome size of 160–180 bp [22]. Tissue-of-origin deconvolution is a key technique in cfDNA methylation analysis, allowing scientists to determine the source or origin of cfDNA based on DNA methylation patterns [23]. This technique is based on a core concept: Different tissues and organs have unique epigenetic signatures in their DNA methylation profiles. Therefore, when these tissues or organs are damaged, such as due to cancer, trauma, or other diseases leading to cell death, they release cfDNA into the bloodstream (Fig. 1A) [24, 25]. Scientists can analyze the DNA methylation patterns in these cfDNA samples and attempt to compare them with a reference database of known tissue-specific DNA methylation patterns. Through this comparison, they can deconvolve or determine the source tissue or organ of a given cfDNA sample [26]. The advantage of this technique is that it enables scientists to detect and monitor tissue-specific damage in a non-invasive manner, without the need for traditional tissue biopsies.

Hence, the release of epigenetic markers in the form of cfDNA into the bloodstream following tissue and organ damage, as illustrated in Fig. 1B, makes cfDNA a valuable biomarker for assessing tissue and organ damage [27]. Furthermore, the epigenetic state of cfDNA, including DNA hypermethylation and hypomethylation patterns, can provide insights into the extent of tissue and organ damage [14]. Overall, the DNA methylation state of cfDNA can be used as a biomarker for tissue and organ damage. In this review, we will examine the underlying mechanisms involved, the current research status of cfDNA methylation pattern for detecting organ and tissue damage, as well as potential applications of these assays in clinical practice.

## Epigenetics and DNA methylation

Eukaryotic gene expression is intricately regulated through numerous mechanisms, even when the DNA sequence remains unchanged. This phenomenon is known as epigenetics [28]. Epigenetic regulation involves various mechanisms, including DNA methylation, histone modifications, and the influence of non-coding RNA, among others. Among these, DNA methylation is extensively studied [29].

In eukaryotes, DNA methylation involves adding a methyl group to the 5th carbon of C in DNA, catalyzed by DNA methyltransferases. This creates 5-mC (5-methylcytosine), found mainly at CpG sites [30]. There are about 28 million CpG sites in the human genome, but they are not evenly distributed. CpG sequences occur at only about 1% on average, but CGIs (CpG islands) are densely packed regions with more than five times the average frequency, typically defined as genomic regions over 200 base pairs long, with over 50% GC content, and a CpG ratio exceeding 60% [31]. Over 60% of genes, including those with tissue-specific expression patterns, have CpG islands in their promoter regions and first exons [32].

In normal tissue genomes, about 70% of CpG sites are methylated, while most CpG islands remain unmethylated [33]. Some genes with CGIs are methylated, such as genes on the inactive X chromosome and imprinted genes [34]. Methylation plays a vital role in various biological processes, including cell growth, differentiation, X-chromosome inactivation, genomic imprinting, gene silencing, defense against foreign genes, and xenobiotic metabolism in multicellular organisms.

The study of epigenetic mechanisms holds immense significance in scientific research and medicine, enhancing our understanding of gene regulation, disease occurrence and inheritance, and the development of innovative therapies.

## Mechanisms of DNA methylation change in organ and tissue damage

**Fig. 2:**
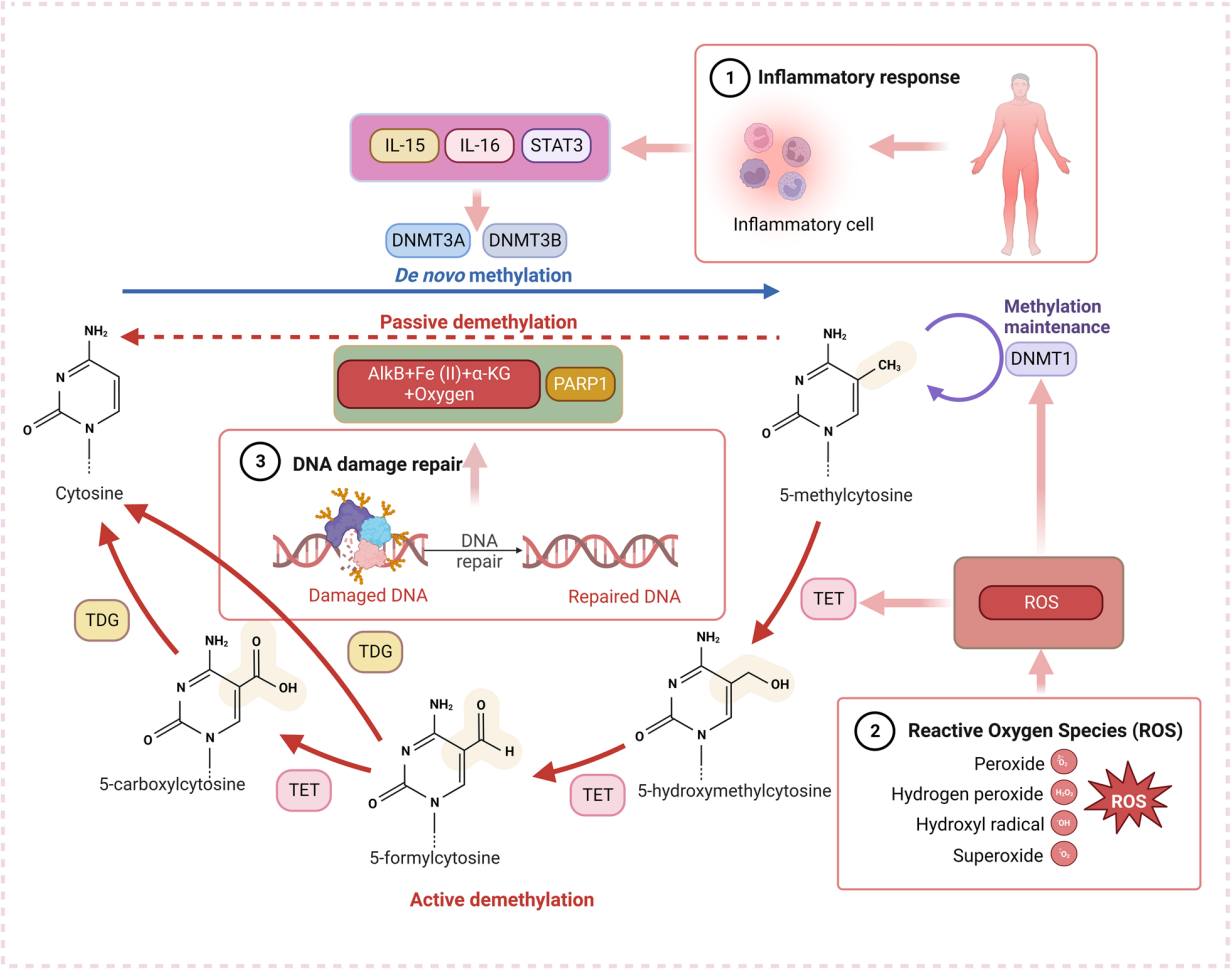
DNA methylation and demethylation mechanisms in organ and tissue damage. Tissue and organ damage can regulate DNMTs and DNA demethylases (e.g., TET and TDG) through various mechanisms, thereby influencing the state of DNA methylation, including inflammation, oxidative stress, and DNA damage repair mechanisms

DNA methyltransferases (DNMTs) are a class of enzymes that play a crucial role in cells, regulating DNA methylation [35]. DNMTs are primarily responsible for adding methyl groups to cytosine residues in DNA, forming 5-methylcytosine, thereby achieving DNA methylation. DNMTs are capable of binding to DNA and identifying target DNA sequences, often CpG. Once DNMTs bind to the target DNA sequence, they transfer a methyl group from the SAM donor to the cytosine base in DNA. This process involves the formation of a covalent bond, adding the methyl group to the fifth carbon atom of the cytosine base, resulting in 5-mC. DNMT1 (DNA methyltransferase-1) serves as a maintenance DNMT primarily tasked with upholding the stability of DNA methylation during DNA replication and cell division. DNMT1 can recognize newly synthesized unmethylated DNA strands and add methyl groups during DNA replication to ensure the correct inheritance of DNA methylation patterns in each generation of cells [35]. DNMT3A and DNMT3B are de novo DNMTs responsible for introducing new DNA methylation during cell differentiation and development. These enzymes can introduce methyl groups into specific genes or genomic regions, thereby regulating gene expression and influencing cell function. DNA demethylation: In addition to DNMTs, DNA demethylases like TET (ten–eleven translocation) enzymes are also crucial components of the dynamic balance of DNA methylation. TET enzymes can remove methyl groups from DNA, converting 5-mC into 5hmC (5-hydroxymethylcytosine), and participate in the DNA demethylation process [36]. AID (activation-induced cytidine deaminase) and AB (Apobec) are deaminases, typically associated with deamination of DNA bases and deoxyribose modification, rather than directly participating in DNA methylation or demethylation reactions [37]. They function in altering DNA bases, such as converting cytosine to uracil, without involving the addition or removal of DNA methyl groups [38]. TDG (thymine DNA glycosylase) and SMUG1 (single-strand-selective monofunctional uracil-DNA glycosylase 1) are two enzymes related to DNA repair, playing a crucial role in maintaining the integrity of DNA bases and correcting base errors in DNA [37]. These enzymes introduce changes such as deamination or deoxyribosylation to DNA molecules, influencing DNA methylation status. Tissue and organ damage can regulate DNMTs and DNA demethylases through various mechanisms, thereby influencing the state of DNA methylation, including inflammation, oxidative stress, and DNA damage repair mechanisms. These mechanisms can interact with each other to cause complex changes in DNA methylation patterns and lead to the continuation of damage and disease (Fig. 2).

### Inflammatory response

After tissue and organ damage, the body initiates an inflammatory response to clear damaged cells and tissue, promoting repair and regeneration [39]. However, this inflammatory response may also cause changes in DNA methylation, as the inflammatory response can produce reactive oxygen species [40], nitrites, and other harmful substances that can directly or indirectly affect DNA methylation. Inflammation also can activate immune cells, leading to the secretion of cytokines and other signaling molecules that can alter DNA methylation patterns. For example, the IL-6 (cytokine interleukin-6) and STAT3 (signal transduction and transcriptional activator 3) have been shown to increase the activity of DNMTs, leading to increased DNA methylation in certain genes [41, 42]. Excessive IL-15 (cytokine interleukin-15) has been shown to induce DNMT3b (DNA methyltransferase-3b) upregulation and global DNA hypermethylation in animal model [43, 44].

### Oxidative stress

Tissue and organ damage may increase cellular oxidative stress due to the action of free radicals, peroxides, and other oxidizing agents released by damaged cells, which can damage DNA and alter the activity of enzymes that regulate DNA methylation. For example, oxidative stress can cause the oxidation of DNMTs, leading to change activity and altered DNA methylation patterns [45–48]. In a recent study on peripheral blood mononuclear cells of Graves patients, it was found that increased reactive oxygen species led to increased expression of DNMT1, which in turn was associated with abnormal methylation patterns of immunoregulatory genes that promote autoimmunity in Graves disease [49]. Similarly, in studies of doxorubicin-induced cardiotoxicity, doxorubicin induces cardiac cell damage and oxidative stress, which produces free radicals and nitric oxide in response to stress. Oxidative stress increases and down-regulates DNMT1 enzyme activity and leads to mitochondrial dysfunction of DNA methylation [50]. Oxidative stress can also alter the activity of enzymes that remove methyl groups from DNA, such as TET enzymes, leading to changes in DNA methylation patterns. In a cell damage model constructed in the study of polycystic ovary syndrome disease, it has been found that cell damage reduces the level of TET and leads to increased levels of cell methylation [51, 52].

### DNA damage repair

DNA damage is a common consequence of tissue and organ damage, and DNA repair mechanisms are activated to repair the damage. The process of DNA repair can affect DNA methylation patterns, as some DNA repair enzymes can modify DNA methylation levels [53, 54]. For example, the enzyme PARP1 (the enzyme poly (ADP-ribose) polymerase-1) is a nuclear enzyme involved in DNA repair, chromatin remodeling and gene expression, which can catalyze the removal of methyl genome-wide chromatin [55]. The binding of PARP1 to chromatin genome-wide is mutually exclusive with DNA methylation pattern suggesting a functional interplay between PARP1 and DNA methylation [56]. In fact, inhibition of PARylation leads to genome-wide changes in DNA methylation patterns. DNA dioxygenase AlkB also can regulate the DNA methylation level, and AlkB can directly converts m3C and m1A into unmethylated bases by the mechanism of oxidative demethylation in the presence of Fe(II) ions, α-KG, and oxygen [57, 58]. In a study of blood diseases, B cells in germinal centers differentiate with the help of DNMT1, changing their DNA patterns in order to differentiate properly. DNMT1 has been shown to play a dual role in DNA methylation and double-strand break repair [59].

In summary, tissue and organ damage can cause changes in DNA methylation patterns through multiple mechanisms, which can interact with each other to create complex changes in DNA methylation patterns.

## 5-mC methylation detection techniques

Researchers utilize various DNA methylation detection methods to study the methylation patterns in cfDNA, thereby understanding methylation changes in both healthy and disease states. These methods mainly encompass a range of 5-mC methylation assays, which can be categorized as follows:

### Methylation detection method without sequencing

Non-sequenced methylation detection methods typically do not involve DNA sequencing but instead rely on various chemical or biological techniques to analyze DNA methylation status. Among these methods are restrictive enzyme digestion, which involves cleaving DNA at specific nucleotide sequences using methylation-sensitive enzymes, allowing for the study of DNA methylation. For instance, methods like von Kanel et al.’s combining restriction enzyme digestion and real-time PCR or Xianfeng Wang et al.’s CRISPR/Cas13a-based strategy enable precise assessment of methylation status and copy number changes at individual sites [60, 61]. Additionally, MSP (methylation-specific PCR) and other PCR-based approaches using bisulfite-treated DNA templates are sensitive and specific techniques for analyzing DNA methylation at specific sites, with MSP selectively amplifying methylated or unmethylated DNA sequences using methylation-specific primers [62]. These methods, including TaqMan-real-time FQ-MSP (TaqMan real-time methylation-specific PCR) and ddPCR (digital droplet PCR), offer high precision and sensitivity, making them valuable tools for the study of DNA methylation [63]. For example, MSP combined with ddPCR has been utilized in the detection of bile duct cancer markers in patients with primary sclerosing cholangitis [64]. Furthermore, HRM (high-resolution melting) is a method for detecting DNA methylation based on the distinct melting behavior of methylated and unmethylated DNA fragments. HRM allows for qualitative and quantitative analysis of DNA methylation without the need for costly sequencing techniques, providing a rapid, high-throughput, and cost-effective approach widely used in DNA methylation research. The MS-HRM (methylation-sensitive high-resolution melting) protocol by Wojdacz and Dobrovic can detect methylated templates in an unmethylated background with sensitivity comparable to MSP [65, 66]. Lasse Kristensen et al. also developed a probe-free quantitative MSP assay using HRM analysis for reliable detection, even at 0.1% methylated standards [67]. Additionally, methylation arrays such as Illumina's HM450K (Illumina Infinium HumanMethylation450 BeadChip) and 850K (Illumina Infinium Methylation EPIC Bead Chip) offer cost-effective and high-throughput platforms for comprehensive methylation analysis, with HM450K targeting over 485,000 cytosine positions across the human genome and being widely used in cancer research [68, 69]. The newer 850K array extends coverage, including additional probes targeting regulatory regions, but these arrays have limited genome-wide coverage and may not capture all methylation information [70].

### Methylation detection method with sequencing

DNA methylation detection methods based on sequencing encompass a range of techniques used to study DNA methylation patterns. One widely used method is BSP (Bisulfite sequencing PCR), which combines PCR amplification with Sanger sequencing to identify methylation sites at individual CpG sites [71]. BSP comes in two forms: direct BSP, which directly sequences PCR products, and cloning BSP, which involves cloning PCR products into vectors for sequencing [72, 73]. However, BSP has limitations, such as sensitivity to low-level mosaicism and potential PCR-induced bias.

Another powerful technique is Pyrosequencing, which enables high-resolution and sensitive monitoring of DNA methylation in real time by quantitatively measuring the incorporation of nucleotides [74], which makes it valuable for various research and clinical applications, including cancer prediction and repetitive element investigation [75, 76].

WGBS (whole-genome bisulfite sequencing) provides comprehensive and high-resolution genome-wide methylation information, but it comes with challenges such as complex data processing and substantial sequencing requirements [77, 78]. MCTA-seq (methylated CpG tandems amplification and sequencing), on the other hand, is a sensitive method capable of analyzing minute genomic DNA amounts (as low as 7.5 pg), showing promise for non-invasive cancer screening [79]. However, it may require specialized equipment and offers limited coverage compared to whole-genome methods. Targeted bisulfite sequencing selectively amplifies specific genomic regions, combining the cost-effectiveness of methylation arrays with bisulfite sequencing's digital signal output [80, 81]. It can be tailored for specific genes or regions but may require additional time and resources for probe design and synthesis [82]. RRBS (reduced representation bisulfite sequencing) is a cost-effective approach that focuses on CpG-rich regions and has enabled advancements like scRRBS (single-cell RRBS), offering insights into tumor burden estimation and disease biomarker discovery [83, 84]. MeDIP-Seq (methylated DNA immunoprecipitation sequencing) reduces the required DNA input for methylation sequencing, making it suitable for cfDNA methylation detection [85, 86]. An extension of this method, cfMeDIP-seq (cell-free DNA-methylated DNA immunoprecipitation and high-throughput sequencing), can be applied to various sample types with limited DNA availability, potentially extending its applications beyond cancer detection [87–90]. MBD-Seq (CpG methylation binding domain enrichment sequencing) uses MBD (methyl-CpG binding domain) proteins to selectively capture methylated DNA fragments, effectively identifying and quantifying methylated regions, particularly in CpG-rich regions and CGIs [91, 92]. An ultra-low input cfDNA methylation analysis method, cfMBD-seq (cfDNA MBD-Seq), optimizes MBD capture conditions for use in various sample types [93].

Nanopore sequencing is a revolutionary technology that directly measures electrical current changes as DNA molecules pass through nanopores, enabling direct methylation detection without the need for chemical modifications [94, 95]. For example, ONT (Oxford Nanopore Technologies), a company that provides DNA sequencing platforms based on Nanopore technology [96, 97]. This technology offers advantages such as high throughput, high resolution, and single-molecule sequencing, making it valuable in various research and clinical applications, including cancer detection [98]. However, it has limitations, including higher per-base cost and error rates for single-nucleotide variants and insertions/deletions [99, 100]. PacBio sequencing, based on SMRT (single-molecule real-time) technology, generates exceptionally long DNA read lengths, making it suitable for studying complex genomes, structural variations, repetitive sequences, and epigenetic changes in repetitive elements [101, 102]. It is versatile in analyzing diverse genomes and DNA samples without GC or AT content preferences [103].

## Research status of cfDNA methylation detection of specific tissue and organ injury

cfDNA methylation-based detection has great potential in the detection of tissue and organ damage, and further research in this field can provide new diagnostic and prognostic tools for various tissue and organ diseases. The following describes the current state of research of cfDNA methylation in the detection of tissue and organ damage in specific organ (Table 1).

**Table 1:** cfDNA methylation markers of specific tissue and organ injury

Organ	Disease	Observations	Detection method	Sensitivity	Specificity	References
Liver	Acute graft-versus-host disease	PTK28	ddPCR	92.8%	91.0%	[104]
Liver	Hepatocellular carcinoma	cg02396797, cg030646442, cg21178851	Bisulfite DNA sequencing and HM450K	35%	95%	[27]
Liver	Acute hepatocyte death	ITIH4, IGF2R, VTN	HM450K and ddPCR	–	–	[105]
Colon	Acute graft-versus-host disease	SESN3	ddPCR	90.5%	98.9%	[104]
Kidney	Acute kidney injury	KLK1	Pyrosequencing	–	–	[106]
Kidney	kidney transplant	CALCA	Fluorescence-based real-time PCR	–	–	[107]
Kidney	Kidney transplant	Absolute concentration of kidney cfDNA	WGBS	–	–	[108]
Heart	Myocardial infarction	CORO6	MCTA-seq and ddPCR	46%	80%	[109]
Heart	Open-heart surgery damage	Cardiac cfDNA concentration	Two-step multiplexed PCR and NGS	–	–	[110]
Heart	Acute myocardial infarction	FAM101A	WGBS and MSP sequencing analysis and ddPCR	–	–	[111]
Lung	Lung cancer and lung nodules	cg19864007_cg22636429_cg15542994, cg26970841_cg03978375_cg24826867, cg04175417, cg21962423, cg23156742, cg06287318, cg21963643, cg07568344 and cg12545252	Targeted methylation sequencing	–	93.2%	[112]
Pancreas	Diabetes	Fbxl19, Mtg1, Leng8, Zc3h3, INS, INS antisense	HM450K and two-step multiplex PCR	–	70%	[113]
Pancreas	Diabetes	CHTOP/INS	HM450K and bisulfite DNA sequencing and ddPCR	–	100%	[114]
Brain	Oligodendrocyte	cg26765599, cg20637405, cg25396488	Bisulfite DNA sequencing and HM450K	20.7%	95%	[27]
Brain	Neuron	cg13131859, cg10030512, cg12560421, cg18519737	Bisulfite DNA sequencing and HM450K	17.2%	95%	[27]
Brain	Astrocyte	cg22031783, cg01623475, cg01623475	Bisulfite DNA sequencing and HM450K	13.8%	95%	[27]
Muscle	Amyotrophic lateral sclerosis	RHBDF2	Pyrosequencing and bisulfite cloning sequencing	–	–	[115]

### Liver

Liver-derived cfDNA methylation-based assays have been used to detect liver damage in various contexts, including hepatitis B and C infections, non-alcoholic fatty liver disease, hepatocellular carcinoma, and liver transplantation. Miguel Waterhouse et al. used digital droplet PCR to measure PTK28 gene-specific methylation markers in cfDNA to detect liver tissue damage in patients with acute graft-versus-host disease [104]. Thresholds to differentiate aGvHD (acute graft-versus-host disease) from non-aGvHD (non-acute graft-versus-host disease) in liver 1.5 (sensitivity = 0.928; specificity = 0.910). And clinical improvement of liver damage aGvHD resulted in methylated PTK2B reduced concentration. In another study of cancer damage to host tissue, it was found that cell death in organs affected by cancer could be detected by tissue-specific methylation patterns of cfDNA. In the study of hepatocellular carcinoma, three liver tissue methylation-specific markers (cg02396797\cg030646442\cg21178851) were identified by Illumina Infinium 450k microbead array [27]. Patients with liver metastases exhibited higher levels of hepatocyte-derived cfDNA, measured either by the fraction of cfDNA derived from hepatocytes or the hepatocyte genome equivalents per milliliter, compared to healthy donors, patients with local cancer, or patients with non-liver-metastatic disease. Using a cutoff of 561 (genome equivalents/mL) of liver-derived specific cfDNA markers, the specificity and sensitivity for detecting liver metastases and liver damage were 95% and 35%, respectively [27]. Moreover, hepatocyte cfDNA levels were able to distinguish patients with stage 4 cancer with and without liver metastases (AUC = 0.81, 95% CI 0.73–0.89, *P* < 0.0001). Another study described a method for detecting acute hepatocyte death by identifying three genomic sites (ITIH4, IGF2R and VTN) that are specifically non-methylated in hepatocytes based on quantification of cfDNA fragments carrying hepatocyte specific methylation patterns [105]. Blood samples from healthy individuals, liver transplant patients, liver donors, sepsis patients, and Duchenne muscular dystrophy were tested by ddPCR. The results showed that these three measurements of hepatocellular derived cfDNA can provide specific and sensitive information about hepatocellular death. It provides a near real-time indication of liver damage and can monitor liver damage dynamically.

### Kidney

Methylation-based assays have been increasingly utilized to detect kidney damage and understand the underlying molecular mechanisms associated with kidney diseases. Hypermethylation of specific gene promoter regions has been observed in kidney patients. A study of human acute kidney injury, the methylation status of four sites in the promoter region of cfDNA KLK1 gene was evaluated by pyrosequencing in urine and whole blood of healthy controls and acute kidney injury patients [106]. The results showed that the methylated KLK1 gene promoter in blood and urine of AKI patients was higher than that of healthy controls in global genomic patterns, and *P* < 0.0001, indicating that KLK1 gene methylation has the potential to be a marker for monitoring kidney injury. In another study, abnormal methylation of two gene promoters (DAPK and CALCA) in the urine DNA of 13 deceased and 10 living kidney transplant recipients and 65 healthy controls was detected by quantitative methylation-specific polymerase chain reaction on the second day after surgery, and CALCA was found in the urine of transplant recipients Gene promoters were significantly more likely to be abnormally hypermethylated than healthy controls (100% vs. 31%; *P* < 0.0001) [107]. CALCA hypermethylation was increased in urine of deceased patients compared with living donor transplantation (21.60 ± 12.5 vs. 12.19 ± 4.7; *P* = 0.04). In addition, urine CALCA abnormal hypermethylation tended to increase in patients with biopsy-confirmed acute tubular necrosis compared with acute rejection and slow or rapid graft function (mean: 20.40 ± 6.9, 13.87 ± 6.49, 17.17 ± 13.4; *P* = 0.67) (16.9 ± 6.2 and 18.5 ± 13.7; *P* = 0.5). These results suggest that urine DAPK and CALCA gene epigenetics is a promising method for kidney transplantation of biomarkers of acute ischemic injury. In a study with kidney transplant patients, urine cfDNA was analyzed to understand the relationship between infections and kidney tissue damage [108]. The results showed that after transplant, urine absolute concentration of tissue-specific cfDNA increased due to stress damage but returned to baseline after 10 days without infection. For patients with nephropathy and BK virus infection, nephrogenic cfDNA levels were significantly higher than in normal patients or those with BK virus alone (*P* = 4 × 10^−4^, *P* = 7.9 × 10^−3^). Additionally, bladder and leukocyte cfDNA levels were elevated in patients with bacterial infections (AUC = 0.91), indicating a possible link between infection and tissue damage. And the fragment size profile of cfDNA can provide an additional indicator by which patients with different symptoms of infection can be stratified. These findings suggest that analyzing cfDNA levels could improve our understanding of kidney damage related to infections and potentially lead to better diagnostic tools and treatments.

### Heart

cfDNAs methylation-based assays have been used to detect heart damage in various contexts, including open-heart surgery and MI (myocardial infarction). A new study evaluated six unmethylated sites of cfDNA molecules as markers of cardiac injury after open-heart surgery [110]. The six myocardial cell-specific DNA unmethylated markers were used to measure cardiac cfDNA in plasma of 42 infants undergoing open-heart surgery. Cardiac cfDNA was elevated after surgery, reflecting tissue damage directly associated with surgery, and decreased in most patients after surgery. This study also used cardiac cfDNA levels to predict surgical outcomes, selected duration of mechanical ventilation (greater than or less than 24 h) and maximum VIS (greater than or less than 20 h), and used ROC (receiver operating characteristics) curves as clinical outcomes. As a predictor of mechanical ventilation duration or maximum VIS, the AUC of cardiac cfDNA at 6 h after surgery was 0.7475 and 0.7555, respectively. Similarly, a cardiomyocyte methylation marker (FAM101A) was identified in another study [111]. ddPCR was used to measure plasma cfDNA concentration of fully unmethylated FAM101A, which can sensitively and specifically detect cardiomyocyte injury. Clinical observations in sepsis patients have shown that the level of cardiac cfDNA is not significantly affected by kidney or liver damage [111]. Jie Ren et al. found that when myocardial damage occurs, cfDNA is released and identified six CGCGCGG loci that were located in the CGIs of CORO6, CACNA1C (two loci), OBSCN, CRIP1 and ZNF503-AS2, showing heart-specific hypermethylation patterns [109]. Among these markers, CORO6 showed the most specific methylation pattern in the heart. As a result, they proceeded to investigate the development of a ddPCR assay for this specific locus. This assay effectively detected signals of heart damage in cfDNA from MI patients upon their admission to the hospital [109]. They therefore explored the development of a ddPCR assay for this locus that clearly detected heart damage signals in cfDNA of MI patients at hospital admission [109].

### Lung

Lung organ specificity cfDNAs methylation-based assays have been used to detect lung damage in various contexts, including chronic obstructive pulmonary disease, lung cancer, lung nodules, and after bronchial testing. In 2022 research, it determined the methylation status of 17 loci with lung-specific methylation patterns, and used it to assess lung-derived cfDNA in the plasma of healthy volunteers and patients with lung disease [116]. The results showed universal cfDNA methylation markers of normal lung epithelium allow for mutation-independent, sensitive, and specific detection of lung-derived cfDNA, reporting on ongoing lung injury. Wenhua Liang et al. developed a new non-invasive diagnostic method based on nine markers in cfDNA methylation analysis released by lung injury to detect early lung cancer and distinguish lung cancer from benign lung nodules and the model achieved a specificity of 93.2% [112]. The nine markers are cg19864007_cg22636429_cg15542994, cg26970841_cg03978375_cg24826867, cg04175417, cg21962423, cg23156742, cg06287318, cg21963643, cg07568344, and cg12545252.

### Brain

In the 2022 liquid biopsy study exploring collateral tissue damage, 10 genomic sites were also identified that were unmethylated in neurons (cg13131859/cg10030512/cg12560421/cg18519737), oligodendrocytes (cg26765599/cg20637405/cg25396488), or astrocytes (cg22031783/cg01623475/cg01623475) [27]. Signals from each brain cell type generate an ROC curve. Markers of each brain cell type were able to identify plasma from patients with brain metastases with an AUC of 0.72–0.81, with a 95% specific sensitivity of 17.2% for neuronal markers, 13.8% for astrocyte marker.

### Pancreas

Methylated tissue studies have been able to locate specific cell types in organs, and cell damage can be detected by cfDNA methylation analysis. For example, in a study of plasma pancreatic beta cell-specific cfDNA, six specific biomarkers (Fbxl19, Mtg1, Leng8, Zc3h3, INS, INS antisense) were found to be completely unmethylated in 70% of beta cells [113]. The remaining 30% showed methylation with one or two CpG sites. And the study found that cfDNA methylation markers in pancreatic beta cells were significantly elevated after islet transplantation, reflecting the damage and death of beta cells. Also, given the increased frequency of unmethylated INS CpG sites in beta cells, the ratio of unmethylated to methylated INS DNA released into circulation after cell death is thought to reflect beta cell death. Similarly, a study identified an intragenic CpG site within the gene encoding the chromatin target of PRMT1 (CHTOP), that exhibits complementary tissue specificity to INS and may be used to increase confidence of detecting islet damage in youth with prediabetes and diabetes [114]. The specificity of the two unmethylated biomarkers combined was up to 100%.

### Muscle

ALS (amyotrophic lateral sclerosis) is a progressive neurodegenerative disease that causes the death of upper motor neurons and lower motor neurons. Currently, there is no established circulating biomarker for ALS. Therefore, it is difficult to monitor disease progression and effectively assess treatment response. The cfDNA provides an opportunity to measure ALS cell death that can fill these gaps. Plasma cfDNA was isolated from 20 ALS patients and 20 controls in one study, and cfDNA was used to identify a novel differentially methylated marker in the RHBDF2 gene in ALS patients compared to controls [115]. The study performed ROC analysis to determine the diagnostic effect of ALS of the RHBDF2 gene. Among them, the RHBDF2 gene has two enhancer regions, namely CpG1 and CpG2. The AUC for CpG1 was 0.724 (CI 0.559–0.888; *P* = 0.017), and the AUC for CpG2  was 0.695 (CI 0.527–0.863; *P* = 0.038). The best tipping point for distinguishing ALS patients and controls was 65.97% for CpG1 (sensitivity = 0.850, specificity = 0.526) and 83.26% for CpG2 (sensitivity = 0.800, specificity = 0.474). In existing studies, an efficient EM (expectation maximization) algorithm CelFiE (CELl Free DNA Estimation via expectation–maximization) was developed for methylated cfDNA, where CelFiE input is WGBS reference data, which allows low coverage and noisy data [117]. In that study, cfDNA was detected from ALS patients and age-matched controls with CelFiE. The overall abundance of cfDNA in ALS and controls showed significant differences. ALS (*n* = 28, mean = 297.2 ± 110.57 pg/ul) and controls (*n* = 25, mean = 218.78 ± 139.17 pg/ul). And differences were found in the estimated skeletal muscle ratio in the ALS group, especially the excess in cases compared to the control group (*P* = 5.02 × 10^−2^). Together, these results suggest that cfDNA is a promising direction to identify the first quantitative biomarker of muscle atrophy and death, a hallmark of ALS [118].

**Fig. 3:**
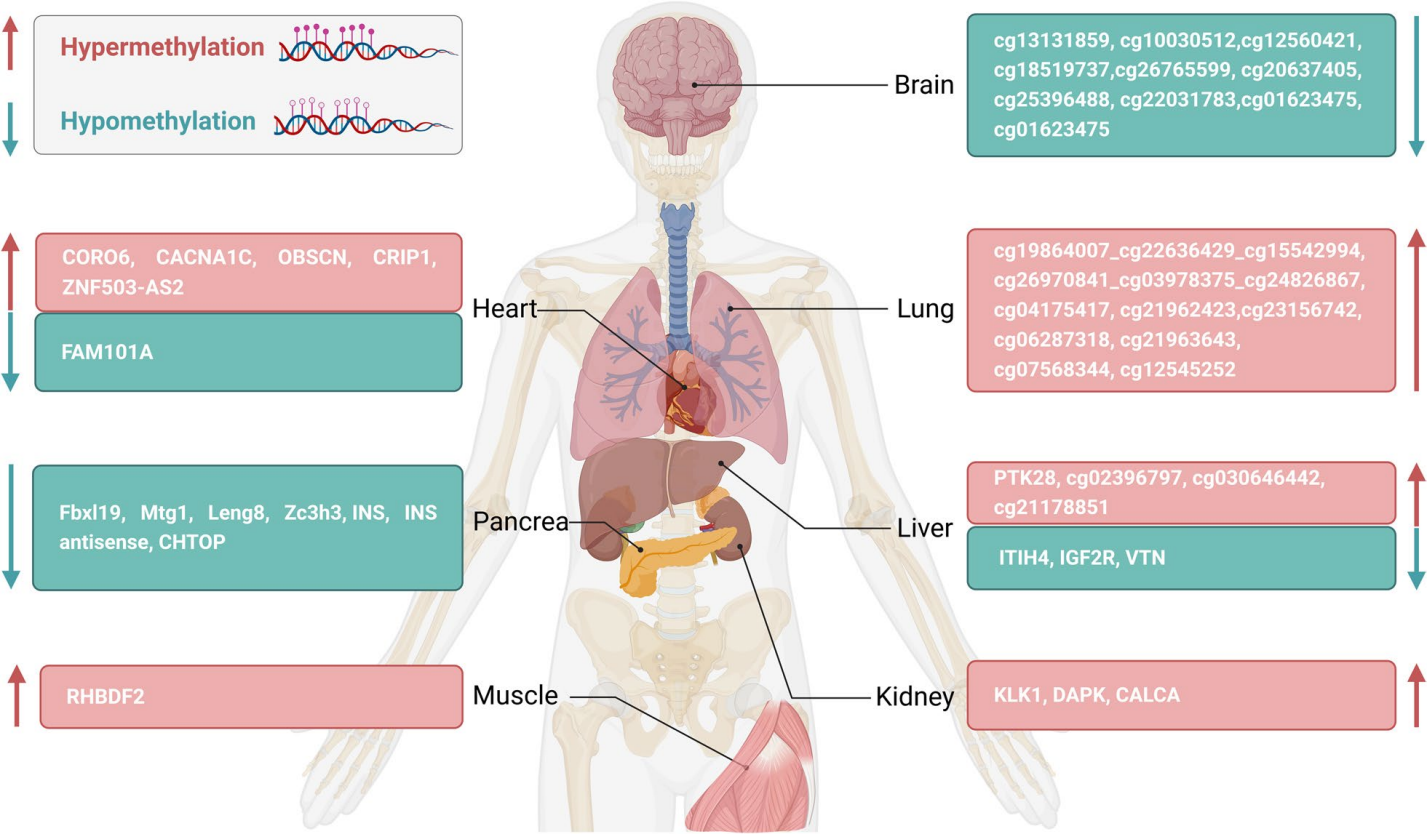
Biomarkers of cfDNA methylation have been discovered in tissue and organ injuries in previous research studies. These biomarkers include 10 related to the brain, 9 to the lungs, 6 to the heart, 7 to the liver, 3 to the kidneys, 7 to the pancreas, and 1 related to muscle

In general, there is a mounting interest in employing methylation-based assays to detect signs of organ and tissue damage, and numerous promising markers have been discovered in various contexts (Fig. 3). However, further research is needed to validate these tissue-specific methylation markers and to develop effective diagnostic and prognostic tools for clinical use.

## The path from the identification of specific cfDNA methylation markers of tissue and organ injury to clinical detection

**Fig. 4:**
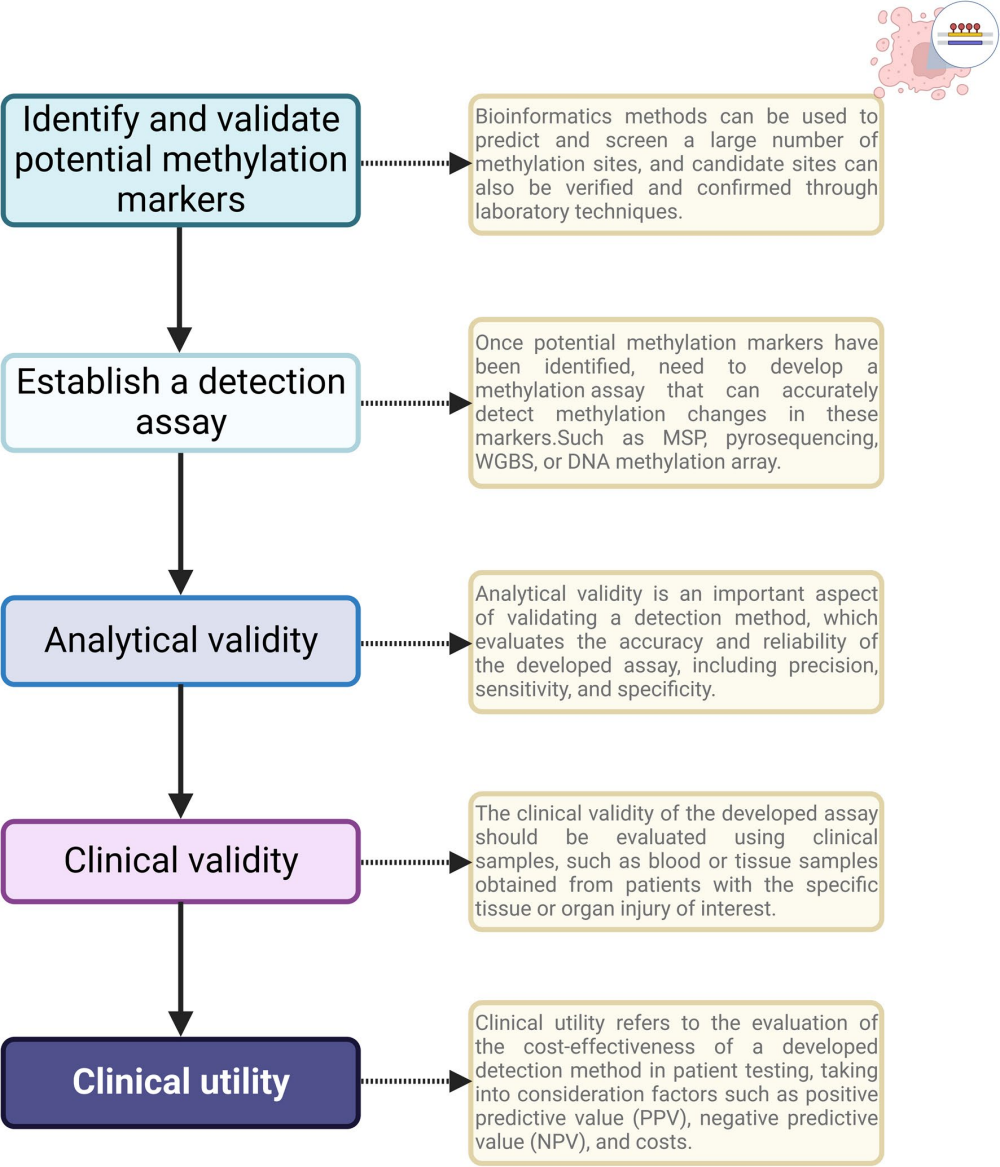
Development path of cfDNA methylation detection for tissue and organ injury. This pathway outlines the progression of research and development necessary to translate potential markers into clinically useful tools for diagnosing and monitoring tissue and organ injuries

Methylation-based damage detection relies on alterations in DNA methylation patterns after the tissue and organ damage. Methylation patterns are unique to each cell type, conserved in the same individual and the same cell type within the individual, and highly stable under physiological or pathological conditions [119]. Therefore, it is possible to utilize specific cfDNA methylation patterns associated with tissue and organ damage to identify the tissue of origin and infer the extent of tissue and organ damage [120]. Identifying particular methylation markers linked to tissue and organ damage and their application in clinical testing necessitates a multi-step approach [121, 122]. However, given the continuous discovery and proposal of new biomarkers, it becomes imperative to establish a robust biomarker verification and certification system to ensure widespread acceptance and proper usage [123, 124]. Adhering to the various principles of biomarker certification, we deduce that the process of identifying specific methylation markers associated with tissue and organ damage and applying them to clinical testing may entail several distinct stages, as illustrated in Fig. 4. Nevertheless, it is important to note that the utilization of methylation analysis for tissue damage detection remains at an early research stage. As of now, no clinical studies have been conducted, and no commercial products have been introduced into clinical settings.

### Identify and validate potential methylation markers

The FDA has four specific categories for contexts of biomarker use: prognostic, predictive, response-indicator, and efficacy-response (Table 2) [123]. Start by conducting a literature review and identifying potential methylation markers associated with tissue and organ injury. This can be done by looking at studies that has investigated methylation changes in response to injury or that have identified DMRs (differentially methylated regions) in diseased tissues compared to healthy tissues. For example, in the study of liver damage biomarkers, hepatocellular specific CpG sites were selected by examining WGBS data and identifying differentially methylated or differentially unmethylated regions [27]. For the selection of hypermethylation markers, they identified regions with a difference exceeding 0.5 between the 75th percentile of hepatocyte samples and the 5th percentile of all non-hepatocyte samples. For hypomethylated markers, a difference of 0.5 between the 95th and 20th percentiles is required. In this way, a total of three hepatocyte markers (cg02396797, cg030646442, cg21178851) were screened. Methylated DNA databases are valuable resources for investigating DNA methylation patterns associated with tissue damage. These databases can be used to identify biomarkers, understand molecular mechanisms, assess severity, monitor treatment response, and study environmental and lifestyle factors. Some of these databases include reference sequence databases, cancer-related databases, and disease-related databases (Table 3). These databases provide valuable resources for researchers interested in identifying methylation markers associated with tissue and organ injury.

**Table 2:** FDA biomarker classification based on contexts of use

Biomarker type	When biomarker is measured	What biomarker indicates
Prognostic	Prior to treatment	Indicates (estimates) the risk or likelihood that a patient who receives no further cancer-directed therapy will experience a specified clinical outcome, such as recurrence, progression, or death
Predictive	Prior to treatment	Interpreted with defined criteria to identify patients who are likely to benefit from a specific treatment compared to patients who do not meet the specified criteria
Response indicator	During or after treatment	Demonstrates a pharmacological or physiological response to the treatment, but does not necessarily signify patient benefit. Examples are declines in prostate-specific antigen, measures of tumor shrinkage, or pharmacodynamic changes in a parameter to show the on-target effect of a drug as proof of mechanism or to optimize dosing
Efficacy response (surrogate)	After treatment	Provides an early and accurate prediction of both a clinical end point, and the effects of the treatment on that end point

**Table 3:** Contents and websites of common DNA methylation databases

Database name	Application	Brief introduction	Website	References
EWAS Data Hub	Reference sequence database	81 tissues/cell types (that contain 25 brain parts and 25 blood cell types), six ancestry categories, and 67 diseases (including 39 cancers)	https://bigd.big.ac.cn/ewas/datahub/	[125, 126]
iMETHYL	Reference sequence database	whole-DNA methylation (~ 24 million autosomal CpG sites), whole-genome (~ 9 million single-nucleotide variants), and whole-transcriptome (> 14,000 genes) data for CD4+ T-lymphocytes, monocytes, and neutrophils collected from approximately 100 subjects	http://imethyl.iwatemegabank.org/index.html/	[127]
MethBank	Reference sequence database	34 consensus reference methylomes, 336 single-base resolution methylomes and/or tissues of five plants, and 18 single-base resolution methylomes from two animals	http://bigd.big.ac.cn/methbank/	[128, 129]
MethCNA	Cancer-related database	10,000 tumor samples representing 37 cancer types	http://cgma.scu.edu.cn/MethCNA/	[130, 131]
MENT	Cancer-related database	It contains DNA methylation, gene expression, correlation of DNA methylation and gene expression in paired samples, and clinicopathological conditions gathered from the GEO and TCGA	http://mgrc.kribb.re.kr:8080/MENT/	[132]
Pubmeth	Cancer-related database	It collects and collates cancer-related methylation data from the literature and manually proofreads and reviews it to provide a high-quality database of cancer-related methylation genes	http://www.pubmeth.org/	[133]
SurvivalMeth	Cancer-related database	It has been developed to investigate the function of DNA methylation-related original effect on the prognosis of cancer, which documented many kinds of DMFEs, including 309,465 CpG island-related elements, 104,748 transcription-related elements, 77,634 repeat elements, and 1,689,653 cell-type-specific super-enhancers and 1,304,902 CTCF-binding regions for analysis	http://biobigdata.hrbmu.edu.cn/survivalmeth/	[134]

### Establish a detection assay

Once potential methylation markers have been identified and need to develop a methylation assay that can accurately detect methylation changes in these markers. The assay could be based on different methodologies such as MSP, pyrosequencing, WGBS, or DNA methylation array, depending on the specific markers being analyzed. And the detection parameters need to be optimized after the analysis method is designed. The assay parameters such as primer design, annealing temperature, cycling conditions, and detection method need to be optimized to ensure accurate and reliable detection of methylation changes in the identified markers.

For example, Miguel Waterhouse developed ddPCR methylation assay based on the methylation of PTK1B and SESN3 genes to analyze tissue damage in patients with acute graft-versus-host disease [104]. Similarly, Tulsi K. Mehta et al. designed primers and probes for specific amplification of CALCA genes to develop a qPCR (quantitative PCR) assay that is promising for the assessment of acute kidney injury in transplant settings [107]. In the study of human acute kidney injury, they evaluated four consecutive CpG methylations of KLK1 gene by pyrosequencing, located between − 203 and − 135 bp from the transcription start site [106]. In the study of cfDNA methylation, Roni Lehmann-Werman et al. established ddPCR program to detect the methylation status of ITIH4, IGF2R and VTN in cfDNA, in which methylation-sensitive TaqMan probe was used to interrogated bisulfite-treated cfDNA [105]. The limited length of the probes (up to 30 bp) determines that they can only cover 2–4 CpG sites that provide information. In the IGF2R locus, four CpGs were covered. However, only two CpGs were covered by probes in VTN = loci, predicting a relatively high frequency of noise (positive droplets) in DNA from non-liver tissue. Two TaqMan probes were designed to increase specificity, each of which identified methylation deficiencies in different pyrimidine clusters (each containing two CpG sites) from the same amplified 100-bp fragment from VTN loci and each probe is labeled with a different fluorophore.

### Analytical validity

Analytical validity is an important aspect of validating a detection method, which evaluates the accuracy and reliability of the developed assay, including precision, sensitivity, and specificity. To ensure the analytical validity of the developed assay for methylation detection, the following steps should be taken: Precision evaluation: Using control samples with known methylation status, assess the precision of the detection method. Calculate internal precision and external precision by analyzing replicate samples to determine the precision of the method. Sensitivity evaluation: Using samples with known lower methylation levels or smaller methylation changes, assess the sensitivity of the detection method. Evaluate the ability of the method to accurately detect small methylation changes. Specificity evaluation: Using samples with different methylation patterns or from different sources, assess the specificity of the detection method. Determine the ability of the method to accurately detect methylation changes without false positives. Validating the developed assay using control samples with known methylation status helps establish the accuracy and sensitivity of the detection method. This assists in evaluating the performance of the method in methylation detection and provides a reliable foundation for its clinical application.

### Clinical validity

After analytical validity has been established, clinical studies are initiated to establish clinical validity: the demonstration that the biomarker and assay are fit for purpose for the specific context of use—that is, that the results will inform the medical decision [135]. The clinical validity of the developed assay should be evaluated using clinical samples, such as blood or tissue samples obtained from patients with the specific tissue or organ injury of interest. This validation process aims to determine whether the assay can effectively distinguish between patients with and without the injury, and assesses clinical sensitivity, clinical specificity, and detection rate in individuals with the injury. Clinical sensitivity refers to the ability of the assay to correctly identify individuals with the specific tissue or organ injury, while clinical specificity refers to the ability of the assay to correctly identify individuals without the injury. The assay should be evaluated for both sensitivity and specificity to determine its accuracy in clinical settings. Additionally, the detection rate in individuals with the injury should be assessed, which reflects the proportion of positive results among patients who have been clinically diagnosed with the injury. This information can help assess the performance of the assay in detecting the specific tissue or organ injury in the intended patient population.

For example, ddPCR methylation assay based on the PTK1B gene was clinically validated in 28 aGvHD patients and 11 patients with no associated symptoms, and the optimal threshold for distinguishing aGvHD from non-AgVHD in the liver was 1.5 (logPTK2 copies /ml plasma; sensitivity: 0.928; specificity: 0.910) [104]. Similarly, Asael Lubotzky et al. recruited 65 healthy donors, 85 patients with local cancer (extrahepatic), 55 patients with metastatic cancer that did not involve the liver, and 63 patients with cancer with liver metastasis to validate the assay based on cfDNA PCR-sequencing analysis of the performance of distinguishing liver metastases. The results showed that the AUC concentration of hepatocytes detected by this method was 0.81 (95% confidence interval = 0.74–0.87, *P* = 0.0001). The specificity and sensitivity of liver metastasis detection are 95% and 35%, respectively.

### Clinical utility

Clinical utility refers to the evaluation of the cost-effectiveness of a developed detection method in patient testing, taking into consideration factors such as PPV (positive predictive value), NVP (negative predictive value), and costs. The evaluation of clinical utility can consider multiple factors, including but not limited to the following.

Diagnostic accuracy: Assessing the sensitivity, specificity, PPV, and NPV of the methylation detection method in correctly identifying tissue or organ injury in patients. Clinical impact: Evaluating the impact of the methylation detection method on treatment decision making and patient management, such as whether it helps in selecting appropriate treatment options, monitoring treatment response, or predicting disease recurrence. Cost-effectiveness: Considering the costs associated with the methylation detection process, including sample collection, laboratory testing, data analysis, and result interpretation, and weighing them against the potential benefits, such as improved patient outcomes, reduced healthcare costs, and enhanced patient satisfaction. Feasibility and scalability: Assessing the practical feasibility of implementing the methylation detection method in a clinical setting, including factors such as ease of sample collection, laboratory infrastructure and expertise required, and scalability to a larger patient population. Comparative effectiveness: Comparing the methylation detection method with existing standard diagnostic methods or alternative approaches in terms of accuracy, clinical impact, and cost-effectiveness. Ethical and legal considerations: Taking into account ethical and legal considerations, such as patient privacy, informed consent, and compliance with regulatory requirements, in the evaluation of clinical utility.

In summary, identifying specific methylation markers associated with tissue and organ injury and applying them to clinical detection requires a rigorous and systematic approach that involves several phases of research, validation, and testing. Nonetheless, it is worth noting that the use of methylation analysis for tissue damage detection is currently in the early stages of research, and as of now, no clinical studies have been conducted, nor have any commercial products been implemented in clinical settings.

Collaboration between researchers, clinicians, and industry partners is necessary to ensure the successful development and implementation of methylation-based diagnostic assays for clinical use.

## Challenges and future directions

Combined with the previous discussion, there are some challenges in studying the application of methylation markers in tissue and organ injury. Challenges and future research directions include:

### Variability in methylation patterns between individuals

According to a large number of previous studies, DNA methylation patterns vary greatly among individuals and are related to gender, living environment and many other factors [136]. Therefore, there are still many difficulties in using DNA methylation analysis to detect tissue damage in clinic. One potential solution to this challenge is to use large-scale studies to identify methylation patterns that are consistent across populations, rather than relying on individual markers [137, 138]. This approach can help identify methylation patterns that are common across different individuals and can be used as a reference for identifying changes in methylation associated with different tissue or organ. By identifying these common patterns, researchers can reduce the impact of individual variability and improve the accuracy of methylation analysis. An alternative approach involves the utilization of machine learning algorithms to discern patterns of methylation that are unique to particular types of tissue damage. This approach moves away from relying solely on individual markers, which can be influenced by individual variability, and instead focuses on comprehensive patterns that can provide more robust and reliable insights [139]. This approach can help identify methylation changes that are associated with specific types of tissue damage or disease. By focusing on specific patterns of methylation, researchers can reduce the impact of individual variability and improve the accuracy of methylation analysis.

### The influence of external factors on methylation patterns

Due to the influence of external factors on DNA methylation patterns, the influence can interfere with the decision making of tissue and organ damage screening, diagnosis and treatment [140–142]. Future research can focus on identifying and controlling for external factors that may influence methylation patterns, such as lifestyle factors and environmental exposures. This can be done through large-scale population studies that collect detailed information about these factors, as well as experimental studies that use animal models to investigate the effects of specific exposures on methylation patterns. For example, when Kang Li studied the relationship between HBV and related chronic hepatitis and the methylation of the peripheral immune system, it was found that the methylation pattern of the peripheral immune system was influenced by external factors. Therefore, they applied Bonferroni correction to the potential confounding factors in the construction of a predictive model for compensatory cirrhosis and found a truly significant correlation [143].

### Technical limitations and inconsistencies in detection methods

Existing methylation detection methods also have technical shortcomings, such as sensitivity and specificity issues. Research in this area can focus on developing more sensitive and specific methods for detecting methylation changes. This can include the use of new technologies such as nanopore sequencing and single-cell sequencing [144, 145], as well as the development of improved bioinformatics tools for analyzing methylation data. Additionally, research can focus on identifying and addressing technical limitations in existing methylation detection methods. For example, bisulfite sequencing, the most commonly used method for detecting DNA methylation, can introduce biases and inaccuracies due to incomplete conversion of unmethylated C and DNA damage during the bisulfite treatment process [146]. New approaches to address these issues, such as the use of alternative chemical treatments or improved enzymatic conversion methods, could improve the accuracy and reliability of methylation detection [147]. Furthermore, research can investigate the impact of technical factors on methylation analysis, such as DNA quality and quantity, sequencing depth, and library preparation methods. Identifying and mitigating these factors can help to reduce variation and increase the reproducibility of methylation studies.

## Conclusion and outlook

Methylation-based assays have shown great potential in detecting organ and tissue damage in a variety of contexts. These assays hold promise for detecting early damage, monitoring the progression of tissue and organ damage, evaluating treatment efficacy and predicting transplant outcomes. However, there are still some challenges to overcome, including variability in methylation patterns, external factors that can influence methylation patterns, and technical limitations in detection methods. Despite these challenges, there is significant interest in this field, and ongoing research continues to identify promising markers and develop effective diagnostic and monitoring tools. As technology continues to advance and DNA methylation patterns become better understood, methylation-based tests have the potential to significantly improve the diagnosis and management of organ and tissue damage in clinical Settings by analyzing blood or other body fluids, which will significantly improve patients' access to diagnostic testing and monitoring. The use of tests based on DNA methylation to predict response to different treatments enables a personalized medical approach to organ and tissue damage. Studying the role of epigenetic changes, including DNA methylation, in the development and progression of organ and tissue damage will also provide insights into disease mechanisms and potential new therapeutic targets.

## Data Availability

The datasets used during the current study are included in this published article and its supplementary information files.

## Abbreviations

cfDNA: Cell-free DNA
C: Cytosine
5-mC: 5-Methylcytosine
CGIs: CpG islands
DNMTs: DNA methyltransferases
SAM: S-adenosylmethionine
DNMT1: DNA methyltransferase-1
TET: Ten-eleven translocation
AID: Activation-induced cytidine deaminase
AB: Apobec
TDG: Thymine DNA glycosylase
SMUG1: Single-strand-selective monofunctional uracil-DNA glycosylase 1
5hmC: 5-Hydroxymethylcytosine
IL-6: Cytokine interleukin-6
STAT3: Signal transduction and transcriptional activator 3
IL-15: Cytokine interleukin-15
DNMT3b: DNA methyltransferase-3b
PARP1: The enzyme poly (ADP-ribose) polymerase-1
MSP: Methylation-specific PCR
TaqMan-real-time FQ-MSP: TaqMan real-time methylation-specific PCR
ddPCR: Digital droplet PCR
HRM: High-resolution melting analysis
MS-HRM: Methylation-sensitive high-resolution melting
EWASs: Epigenome-wide association studies
HM450K: Illumina Infinium HumanMethylation450 BeadChip
NGS: Next-generation sequencing
850K: Illumina Infinium MethylationEPIC BeadChip
WGBS: Whole-genome bisulfite sequencing
MCTA-seq: Methylated CpG tandems amplification and sequencing
RRBS: Reduced representation bisulfite sequencing
scRRBS: Single-cell RRBS
digital-scRRBS: Digital microfluidics-based single-cell reduction representation bisulfite sequencing
MeDIP-Seq: Methylated DNA immunoprecipitation sequencing
cfMeDIP-seq: Cell-free DNA-methylated DNA immunoprecipitation and high-throughput sequencing
MBD-Seq: CpG methylation binding domain enrichment sequencing
MBD: Methyl-CpG binding domain
cfMBD-seq: CfDNA MBD-Seq
ONT: Oxford Nanopore Technologies
SMRT: Single molecule real time
aGvHD: Acute graft-versus-host disease
non-aGvHD: Non-acute graft-versus-host disease
ROC: Receiver operating characteristics
MI: Myocardial infarction
ALS: Amyotrophic lateral sclerosis
EM: Expectation maximization
CelFiE: CELl free DNA estimation via expectation–maximization
DMRs: Differentially methylated regions
qPCR: Quantitative PCR
PPV: Positive predictive value
NVP: Negative predictive value
